# Evaluating Multiple Input Strategies of Large Language Models for Gallbladder Polyps on Ultrasound: Comparative Study

**DOI:** 10.2196/71178

**Published:** 2025-12-23

**Authors:** Lin Jiang, Jiaqian Yao, Zebang Yang, Fuqiu Tang, Xin Zheng, Xiaoer Zhang, Xiaoyan Xie, Ming Xu, Tongyi Huang

**Affiliations:** 1 Department of Medical Ultrasonics Institute of Diagnostic and Interventional Ultrasound The First Affiliated Hospital, Sun Yat-sen University Guangzhou China

**Keywords:** large language models, gallbladder polyps, ultrasound, diagnostic accuracy, medical image analysis, artificial intelligence

## Abstract

**Background:**

Gallbladder polyps have a high prevalence and are predominantly benign lesions, often detected via ultrasound. They impose diagnostic burdens on radiologists while generating substantial patient demand for report interpretation. Benign polyps include nonneoplastic polyps without malignant potential and premalignant adenomas that require cholecystectomy. Current guidelines recommending surgery for polyps ≥1.0 cm may lead to unnecessary interventions. Advanced multimodal large language models (LLMs) such as ChatGPT-4o (OpenAI) and Claude 3.5 Sonnet (Anthropic PBC) demonstrate emerging capabilities in medical image analysis. Implementing LLMs in gallbladder polyp ultrasound evaluation can potentially alleviate radiologists’ workload, provide patient-accessible consultation platforms, and even reduce overtreatment.

**Objective:**

We aimed to analyze the feasibility and conduct an early-stage evaluation of using LLMs for differentiating between adenomatous and nonneoplastic gallbladder polyps (≥1.0 cm) based on ChatGPT-4o and Claude 3.5 Sonnet, compared to assessments by radiologists and the guideline.

**Methods:**

Ultrasound images and reports of gallbladder polyps ≥1.0 cm with pathology were retrospectively collected from a hospital between January 2011 and January 2022. LLM performance was evaluated using three input strategies: (1) direct image analysis (LLMs-image), (2) feature-based text analysis (LLMs-text), and (3) scoring model-based text analysis (LLMs-model). Both intra- and interreader agreement and diagnostic performance of LLMs were evaluated for all three strategies. The diagnostic performance metrics—including sensitivity, specificity, accuracy, area under the receiver operating characteristic curve, and unnecessary resection rate of nonneoplastic polyps of LLMs in the three strategies were compared with the guideline. Additionally, the strategy LLMs-model was specifically compared with radiologists using the same scoring system (strategy readers-model).

**Results:**

This study included 223 patients (aged 18-72 years; 132/223, 59.2% female) as the initial cohort, with 48 adenomatous polyps and 175 nonneoplastic polyps. The external test set comprised 100 patients. The intrareader agreement coefficients for strategy LLMs-model were significantly higher than those for strategy LLMs-image and LLMs-text (all *P*<.01). The interreader agreement of the three diagnostic strategies was ranked as LLMs-model>LLMs-text>LLMs-image. The sensitivity of strategies LLMs-image and LLMs-text was significantly lower than that of the guideline (all *P*<.001). When applying a scoring model (readers/LLMs-model strategy), both radiologists and the LLMs achieved a significantly higher accuracy compared to the guideline (0.34, 0.35, and 0.34 vs 0.22, all *P*<.01), and the unnecessary resection rate of nonneoplastic polyps was significantly lower (82%, 83%, and 83% vs 100%, all *P*<.01), while the sensitivity was comparable to the guideline (0.94, 0.98, and 0.98 vs 1.00, all *P*>.05). All diagnostic performance indicators for GPT-model and Claude-model were not significantly different from those of radiologists (all *P*>.05).

**Conclusions:**

The ability of LLMs to recognize and interpret medical images requires further improvement. The text strategy with a scoring system is currently the most appropriate diagnostic strategy for LLMs.

## Introduction

Large language models (LLMs) are deep learning models trained on large amounts of text data, and their emergence has led to changes in many fields [1-3]. LLMs can understand and generate human language and have the potential to provide medical advice, making their application in the medical field of wide interest [4,5]. With the evolution of the LLMs, their capabilities range from simple summarization to complex tasks such as paper writing, medical education, and diagnosis [6-9]. Prior investigations into the diagnostic applications of LLMs have predominantly used two methodological frameworks: (1) diagnostic strategies using narrative textual inputs describing imaging findings [10], or (2) risk stratification approaches requiring LLMs to apply established scoring systems to textualized lesion characteristics [9]. Some LLMs, such as ChatGPT-4o and Claude 3.5 Sonnet, can analyze and interpret images. These two LLMs were developed by different organizations and demonstrated competitive capabilities that positioned them within the global elite of LLMs at the time of our study [11,12], showing they represent the most advanced level of general-purpose LLMs.

Gallbladder polyps are a common finding in abdominal ultrasound examination, with a reported incidence rate of 6.1%-12.1% [13,14]. They impose diagnostic burdens on radiologists and generate substantial patient demand for report interpretation. The management strategy for polypoid lesions of the gallbladder depends on their pathological type. Neoplastic polyps, including gallbladder cancer and precancerous gallbladder adenomas, require cholecystectomy [15,16]. Studies report that 28%-49.5% of gallbladder adenoma may progress to gallbladder cancer [15,17]. Nonneoplastic polyps, including cholesterol polyps, inflammatory polyps, and fibromyoadenoid polyps, rarely become malignant, and follow-up is recommended [18]. Gallbladder carcinoma can be distinguished from other polypoid lesions based on gallbladder wall continuity and contrast-enhanced patterns [19,20]. However, differentiating adenomatous polyps from nonneoplastic polyps remains challenging. Guidelines recommend cholecystectomy for polyps ≥1.0 cm in size [21]. Using these criteria, 27.1%-56% of patients undergoing cholecystectomy for gallbladder polyps are postoperatively diagnosed with nonneoplastic polyps [22,23]. Beyond financial and psychological burdens, this may result in complications that adversely affect quality of life [24,25]. Therefore, it is critical to distinguish neoplastic polyps from nonneoplastic polyps, particularly for lesions ≥1.0 cm. LLMs can potentially reduce the workload of radiologists by generating descriptions based on ultrasound images or risk stratification of gallbladder polyps, and provide medical consults for patients based on ultrasound reports. If LLMs perform better than existing guidelines or radiologists in differentiating gallbladder polyps, they might reduce unnecessary cholecystectomies. Recent studies have demonstrated that ChatGPT-4o and Claude 3.5 Sonnet exhibit superior performance compared to other LLMs in diagnostic tasks involving radiological imaging [26,27], suggesting potential for ultrasound applications. To date, no study has systematically evaluated LLMs’ ability to characterize sonographic features or differentiate benign gallbladder polyps. Gallbladder polyps manifest as nonshadowing protrusions from the gallbladder wall into the anechoic lumen in ultrasound examinations. This anatomically well-defined nature with intuitive spatial localization makes them suitable for assessing LLMs’ capacity in medical image interpretation. Furthermore, current literature lacks methodological comparisons of LLMs’ diagnostic performance across three distinct paradigms: (1) direct image analysis, (2) text-based diagnosis, and (3) scoring-system–based risk stratification using textualized lesion characteristics.

The purpose of this study was to systematically evaluate the feasibility and conduct an early stage evaluation of LLMs across three diagnostic strategies for differential diagnosis of benign gallbladder polyps (≥1.0 cm) based on ChatGPT-4o and Claude 3.5 Sonnet, with comparison to radiologists and the joint guidelines between the European Society of Gastrointestinal and Abdominal Radiology, the European Association for Endoscopic Surgery and other Interventional Techniques, the International Society of Digestive Surgery–European Federation, and the European Society of Gastrointestinal Endoscopy.

## Methods

### Ethical Considerations

This study was approved by the Ethics Committee in our institute, Sun Yat-sen University (No.2016083). Due to the retrospective nature of this study, the requirement for informed consent was waived. This study adhered to the CLAIM (Checklist for Artificial Intelligence in Medical Imaging) [28] for reporting (Table S1 in Multimedia Appendix 1 [29,30]). This study provides a Reproducibility Checklist in Table S2 in Multimedia Appendix 1 to facilitate the replication of our work.

Throughout the interaction with the LLMs, all patient-identifiable information (including names, hospital ID numbers, etc) was strictly removed from the ultrasound images and reports before analysis.

Due to the retrospective design of this study, participants received no compensation.

### Patient Selection

A total of 447 patients with previous imaging findings of gallbladder polyps who underwent cholecystectomy at our institution were retrospectively reviewed. The initial cohort of 312 patients (January 2011 to January 2022) was used to develop the scoring system and to evaluate the performance of the LLMs, while the subsequent cohort of 135 patients (February 2022 to March 2025) served as an external test set for the LLMs-model strategy. The inclusion criteria were as follows: (1) transabdominal ultrasonography was performed in our hospital before cholecystectomy, and (2) there was a definite postoperative pathological diagnosis. The details of the ultrasound examination protocol are shown in Multimedia Appendix 1 (see also Multimedia Appendices 2-6). Given that the differential diagnostic focus for gallbladder polyps predominantly targets lesions ≥1.0 cm, and smaller polyps may be less clearly visualized in ultrasound images, potentially affecting the performance of LLMs, a 1.0 cm threshold was determined as one of the conditions for patient selection. Patients were excluded if they met any of the following criteria: (1) missing ultrasound images; (2) no polypoid lesions detected by the ultrasound examination in our hospital; (3) age <18 years; and (4) size of the largest polyp <1.0 cm. The patients were divided into an adenomatous polyp group and a nonneoplastic polyp group. Patients with both adenomatous polyps and nonneoplastic polyps were classified as having adenomatous polyps.

### Patient Data Collection

Preoperative clinical data of patients were collected, including demographic information, alanine aminotransferase, and aspartate aminotransferase levels. The latest preoperative ultrasound images and reports were collected. Information about polyp size and number, gallbladder wall thickness measurements, and the presence of gallstones was obtained from the reports. Other ultrasound features were evaluated independently by two radiologists (with 2 years and 10 years of experience in abdominal ultrasound, respectively) who were blinded to the patient’s clinical features and pathology results. Discrepancies were discussed by the two doctors to get a consensus. When consensus could not be reached after discussion between the two radiologists, a third radiologist with seventeen years of experience in abdominal ultrasound made the final determination. If the patient had more than one gallbladder polyp, only the largest one was analyzed. In our previous study, we proposed a new index for quantifying polyp morphology, called the polyp morphology ratio (PMR). The details about the definitions of PMR and other ultrasound features are provided in Multimedia Appendix 1.

### Diagnostic Strategies

This study evaluated ChatGPT-4o and Claude 3.5 Sonnet using their standard web interfaces [31,32] between July and September 2024 according to the following strategies. All interactions were conducted without custom code, API calls, or adjustments to default inference parameters. To ensure transparency, all interactions used the platform defaults, and the complete set of prompts is detailed in Multimedia Appendix 1. The prompts input into the two LLMs were the same. Each prompt input does not include expected outcomes or class distributions, nor does it judge the correctness of the LLM’s responses. For the strategy of LLMs-image, LLMs performed diagnoses based on ultrasound images. Before analysis, images were cropped to remove all patient-identifiable information. Additionally, the images were cropped to ensure the gallbladder occupied approximately 30%-40% of the frame, with lesions centered whenever possible while maintaining the structural integrity of both the gallbladder and surrounding tissues. No image enhancement or standardization was applied. The underlying vision capabilities of the LLMs are accessed directly through their vision application programming interface, rather than via a third-party wrapper. The images input into the LLMs were in JPEG format, with both horizontal and vertical resolutions of 96 dpi. The processed 2D gray-scale ultrasound image and color Doppler ultrasound image were input into LLMs, and the LLMs were required to describe the ultrasound characteristics of gallbladder polyps and provide a diagnosis.

For the strategy LLMs-text, LLMs performed diagnoses based on the text of the ultrasound description. Based on the consensus of the two radiologists, the description of the ultrasound characteristics for the gallbladder polyps was organized into a structured report. The structured report was input into the LLMs, and the LLMs were required to give the diagnosis.

For the strategy LLMs-model, LLMs performed diagnoses based on our previously developed diagnostic model for benign gallbladder polyps ≥1.0 cm [33]. Before this study, a multilevel scoring system for the differentiation between gallbladder adenomatous polyps and nonneoplastic polyps was constructed based on the same cases as those in this study (Table S3 in Multimedia Appendix 1). The relevant information was organized into structured text according to the requirements of the scoring system. Then, only the structured text was input into LLMs without the corresponding scores, and the LLMs were required to calculate the total score and indicate the corresponding grade. In addition, based on the consensus of two radiologists, the total score and the corresponding grade of each patient were calculated with the multilevel scoring system (strategy readers-model).

**Figure 1:**
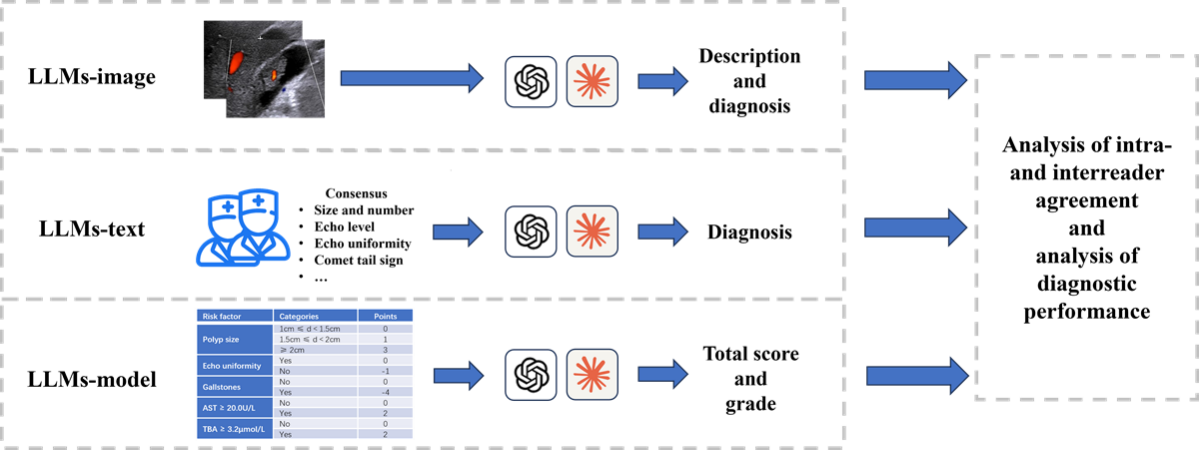
Diagram of three diagnostic strategies for LLMs. LLM: large language model.

Figure 1 shows the flow of the diagnostic strategies for LLMs. The prompts templates in diagnostic strategies for LLMs are in Multimedia Appendix 1. The intrareader agreement, interreader agreement, and diagnostic performance of LLMs in the three diagnostic strategies were evaluated. The initial outputs from LLMs in the three strategies were used to analyze interreader agreement and diagnostic performance. From the final enrolled cases, 70 were randomly selected to assess intrareader agreement. The LLMs were asked to regenerate the output for these cases twice, that is, there were three rounds of output per strategy per LLM for each of these 70 cases, respectively.

### In-Context Learning for LLMs

For the ultrasound image-based lesion characterization tasks in this study, the LLMs underwent supplemental in-context learning beyond their original architecture. LLMs were trained to recognize the degree of blood flow, blood flow pattern, and the definition of PMR and polyp base type (sessile or pedunculated) using ultrasound images of patients with gallbladder polyps in our center (excluding cases included in this study). The prompts used in in-context learning are provided in Multimedia Appendix 1. After entering the prompt word, the memory of the LLMs to was checked to ensure its correct understanding.

### Statistical Analysis

The interclass correlation coefficient, Cohen κ, and weighted κ were used to evaluate the interreader agreement for continuous, unordered, and ordered categorical variables, respectively. Interclass correlation coefficient, Fleiss κ, and Kendall W coefficient were used to evaluate the intrareader agreement for continuous, unordered, and ordered categorical variables, respectively. The interreader agreement of ultrasound features was represented by a heatmap generated with the *pheatmap* package in R (R Foundation). The levels of the coefficient of the agreement analysis were defined as follows: 0.20 or less for slight agreement, 0.21-0.40 for fair agreement, 0.41-0.60 for moderate agreement, 0.61-0.80 for substantial agreement, and 0.81-1.00 for almost perfect agreement. The agreement coefficients were compared using the Z-test. The detailed sample size calculation for intrareader agreement is shown in Multimedia Appendix 1.

The diagnostic performance of LLMs was evaluated by sensitivity, specificity, positive predictive value (PPV), negative predictive value (NPV), accuracy, area under the receiver operating characteristic curve, and unnecessary resection rate of nonneoplastic polyps (UNRR). Sensitivity, specificity, PPV, and NPV were calculated and compared with the *stats* package and *epiR* package in R. The area under the receiver operating characteristic curves were compared using the DeLong test. UNRR was calculated as the number of nonneoplastic polyps recommended for cholecystectomy divided by the total number of nonneoplastic polyps. Chi-square test was used to compare UNRR.

For the strategy LLMs-text, univariate logistic regression was performed on ultrasound features according to the diagnosis of LLMs to analyze the basis for differential diagnosis of LLMs.

Two-tailed *P*<.05 was considered statistically significant. Statistical analysis was performed using PASS 2025 (power analysis and sample size; NCSS, LLC), SPSS 25.0 (IBM Corp), and R version 4.3.1 (R Foundation).

## Results

### Patient Characteristics

**Figure 2:**
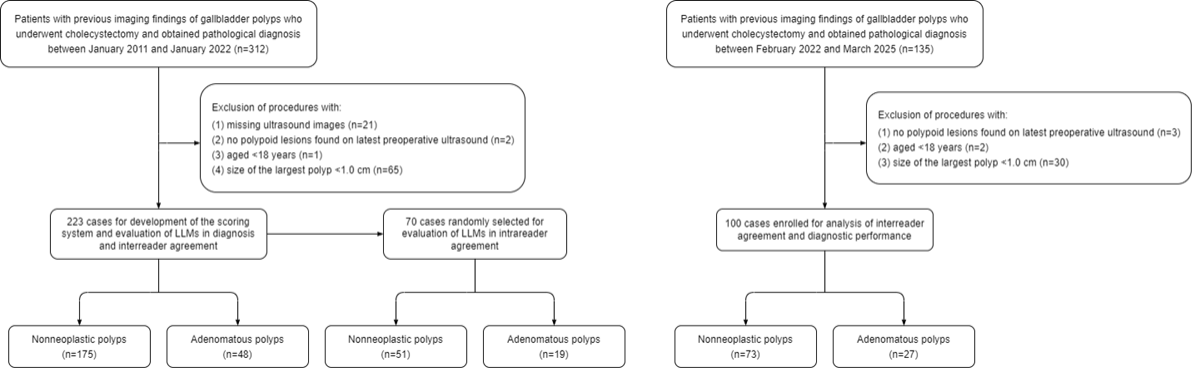
Flowchart of patient selection. LLM: large language model.

In the initial cohort, this study analyzed 223 patients aged 18-72 (median 40, IQR 34-50) years, including 132 (59.2%) females. Among these, 175 (78.5%) had nonneoplastic polyps and 48 (21.5%) had adenomatous polyps. Compared to the initial cohort, the external test set showed no significant differences in demographic characteristics or pathological type distribution (all *P* >.05; Table 1). The patient selection process is shown in Figure 2.

**Table 1:** Demographic characteristics and gallbladder polyp pathology of patients.

Characteristic				Initial cohort (n=223)		External test set for the LLMs^a^-model strategy (n=100)		*P* value
**Sex**								
	Male, n (%)		91 (40.8)		43 (43)		.71	
	Female, n (%)		132 (59.2)		57 (57)		.71	
	**Age (years)**						.84	
		Median (IQR)	40 (34-50)		41 (33-52)			
		Range	18-72		19-74			
**Polyp pathology**								
	Adenomatous polyps, n (%)		48 (21.5)		27 (27)		.28	
	Nonneoplastic polyps, n (%)		175 (78.5)		73 (73)		.28	

^a^LLM: large language model.

### Intrareader Agreement Analysis

The intrareader agreement coefficients of LLMs across three strategies are shown in Table 2. Compared to the intrareader agreement for grades in strategy LLMs-model, both ChatGPT-4o and Claude 3.5 Sonnet exhibited significantly lower intrareader agreement for diagnoses in strategy LLMs-text (0.58 vs 0.97, *P*<.001 for ChatGPT-4o; 0.61 vs 1.00, *P*<.001 for Claude 3.5 Sonnet) and LLMs-image (0.37 vs 0.97, *P*<.001 for ChatGPT-4o; 0.47 vs 1.00, *P*<.001 for Claude 3.5 Sonnet). However, there was no significant difference between strategy LLMs-image and LLMs-text (*P*=.15 for ChatGPT-4o and *P*=.33 for Claude 3.5 Sonnet).

For the ultrasound feature identification in strategy LLMs-image, the agreement level of blood flow degree and pattern for ChatGPT-4o was moderate to substantial (range of agreement coefficient 0.43 to 0.68, 95% CI 0.30 to 0.81). Additionally, there was only slight to fair agreement for ChatGPT-4o in other ultrasound features (range of agreement coefficient –0.12 to 0.30, 95% CI –0.25 to 0.45). However, except for the slight to fair agreement in PMR, comet tail sign and gallbladder wall thickness types (range of agreement coefficient 0.02 to 0.30, 95% CI –0.10 to 0.45), there was moderate to almost perfect agreement for Claude 3.5 Sonnet in other ultrasound features (range of agreement coefficient 0.43 to 0.97, 95% CI 0.30 to 1.00). The intrareader agreement coefficients in ultrasound features for Claude 3.5 Sonnet were all higher than those for ChatGPT-4o, except for PMR. In addition, the intraobserver agreement coefficient in diagnosis for Claude 3.5 Sonnet in strategy LLMs-image was 0.47, which was also higher than 0.7 for ChatGPT-4o.

The intraobserver agreement coefficients in diagnosis for ChatGPT-4o and Claude 3.5 Sonnet in strategy LLMs-text were 0.58, 95% CI 0.46 to 0.72, and 0.1, 95% CI 0.49 to 0.75, respectively, indicating a moderate to substantial level, which were higher than those in strategy LLMs-image.

In strategy LLMs-model, both ChatGPT-4o and Claude 3.5 Sonnet showed almost perfect intraobserver agreement for total score and grade (range of agreement coefficient 0.97 to 1.00, 95% CI 0.94 to 1.00). Almost perfect intraobserver agreement was also observed in the external test set (Table S4 in Multimedia Appendix 1).

**Table 2:** Intrareader agreement coefficients of LLMsa. Data in parentheses are 95% CIs. Except where indicated, the coefficient of agreement analysis is the Fleiss κ coefficient.

			ChatGPT-4o		Claude 3.5 Sonnet
**LLMs-image**					
	PMR^b,c^	0.30 (0.15 to 0.45)		0.02 (–0.10 to 0.14)	
	Echo level^d^	0.25 (–0.03 to 0.42)		0.58 (0.45 to 0.71)	
	Echo uniformity	–0.12 (–0.25 to 0.01)		0.75 (0.62 to 0.88)	
	Comet tail sign	–0.01 (–0.14 to 0.12)		0.30 (0.19 to 0.45)	
	Cauliflower shape	0.00 (–0.13 to 0.12)		0.74 (0.61 to 0.87)	
	Pedunculated or sessile	0.18 (0.05 to 0.31)		0.43 (0.30 to 0.56)	
	Edge	–0.01 (–0.14 to 0.12)		1.00 (—^e^)	
	Blood flow degree^d^	0.68 (0.55 to 0.81)		0.97 (0.94 to 1.00)	
	Blood flow pattern	0.43 (0.30 to 0.56)		0.85 (0.72 to 0.98)	
	Gallbladder wall thickness types	–0.04 (–0.17 to 0.09)		0.15 (0.02 to 0.28)	
	Diagnosis	0.37 (0.24 to 0.50)		0.47 (0.34 to 0.60)	
**LLMs-text**					
	Diagnosis	0.58 (0.46 to 0.72)		0.61 (0.49 to 0.75)	
**LLMs-model**					
	Total score^c^	1.00 (—)		1.00 (—)	
	Grade^d^	0.97 (0.94 to 1.00)		1.00 (—)	

^a^LLM: large language model.

^b^PMR: polyp morphology ratio.

^c^The coefficient of agreement analysis is the interclass correlation coefficient.

^d^The coefficient of agreement analysis is the Kendall W coefficient.

^e^CIs cannot be calculated due to the perfect consistency of the three rounds’ output in each case.

### Interreader Agreement Analysis

**Figure 3:**
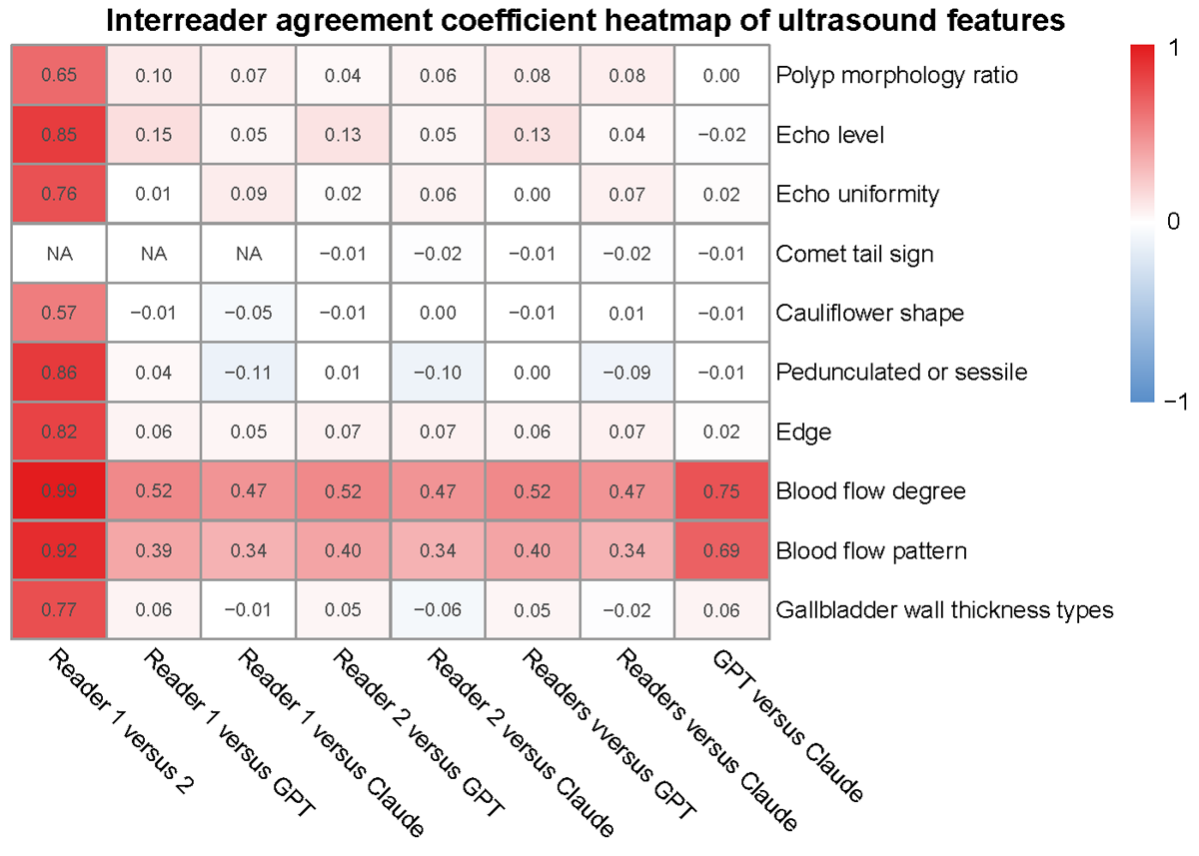
Heatmap of interreader agreement in human readers and LLMs. LLM: large language model.

Figure 3 and Table S5 in Multimedia Appendix 1 present the interreader agreement of human readers and LLMs in ultrasound features. Except for the interobserver agreement level of cauliflower shape between readers 1 and 2 being moderate (agreement coefficient=0.57), the agreement level for other features between readers 1 and 2 was substantial to almost perfect (range of agreement coefficient 0.65 to 0.99). However, the agreement level between LLMs and other observers was only slight (range of agreement coefficient –0.11 to 0.12), except for blood flow degree and pattern (range of agreement coefficient 0.34 to 0.75). As shown in Table S5 in Multimedia Appendix 1, apart from the comet tail sign, the agreement coefficient for other features between readers 1 and 2 was significantly higher than those between LLMs and other observers (*P*<.001).

The agreement coefficient for the comet tail sign could not be calculated because reader 1 considered that the comet tail sign was not present in all cases in this study.

The interobserver agreement coefficients of human readers and LLMs in diagnosis are shown in Table 3. There was slight interobserver agreement between ChatGPT-4o and Claude 3.5 Sonnet in strategy LLMs-image (Cohen κ=0.12, 95% CI –0.01 to 0.24), and fair agreement in strategy LLMs-text (Cohen κ=0.38, 95% CI 0.26 to 0.50). For the strategy readers or LLMs-model, the agreement levels were all almost perfect in readers versus ChatGPT-4o, readers versus Claude 3.5 Sonnet, ChatGPT-4o versus Claude 3.5 Sonnet (range of agreement coefficient 0.84 to 0.99, 95% CI 0.78 to 0.99). In the external test set, LLMs still showed a considerable degree of interreader agreement (Table S6 in Multimedia Appendix 1). Compared to the interreader agreement coefficient of grades between ChatGPT-4o and Claude 3.5 Sonnet in strategy LLMs-model, those for diagnosis in strategy LLMs-image (*P*<.001) and LLMs-text (*P*<.001) were significantly lower. Additionally the interreader agreement coefficient for diagnosis in strategy LLMs-text was significantly higher than that in strategy LLMs-image (*P*=.004).

**Table 3:** Interreader agreement coefficients of human readers and LLMsa in diagnosis. Data in parentheses are 95% CIs. Except where indicated, the coefficient of agreement analysis is Cohen κ. The total score and grade of readers are calculated based on laboratory tests and the US features consensus of readers 1 and 2.

Diagnostic strategies			Readers versus ChatGPT-4o		Readers versus Claude 3.5 Sonnet		ChatGPT-4o versus Claude 3.5 Sonnet
LLM-image			—^b^		—		0.12 (–0.01 to 0.24)
LLM-text			—		—		0.38 (0.26 to 0.50)
**Readers or LLM-model**							
	Total score^c^	0.97 (0.96 to 0.97)		0.98 (0.97 to 0.98)		0.99 (0.98 to 0.99)	
	Grade^d^	0.84 (0.78 to 0.90)		0.95 (0.92 to 0.99)		0.87 (0.82 to 0.92)	

^a^LLM: large language model.

^b^Not available.

^c^The coefficient of agreement analysis is the interclass correlation coefficient.

^d^The coefficient of agreement analysis is weighted κ.

### Diagnostic Performance

**Figure 4:**
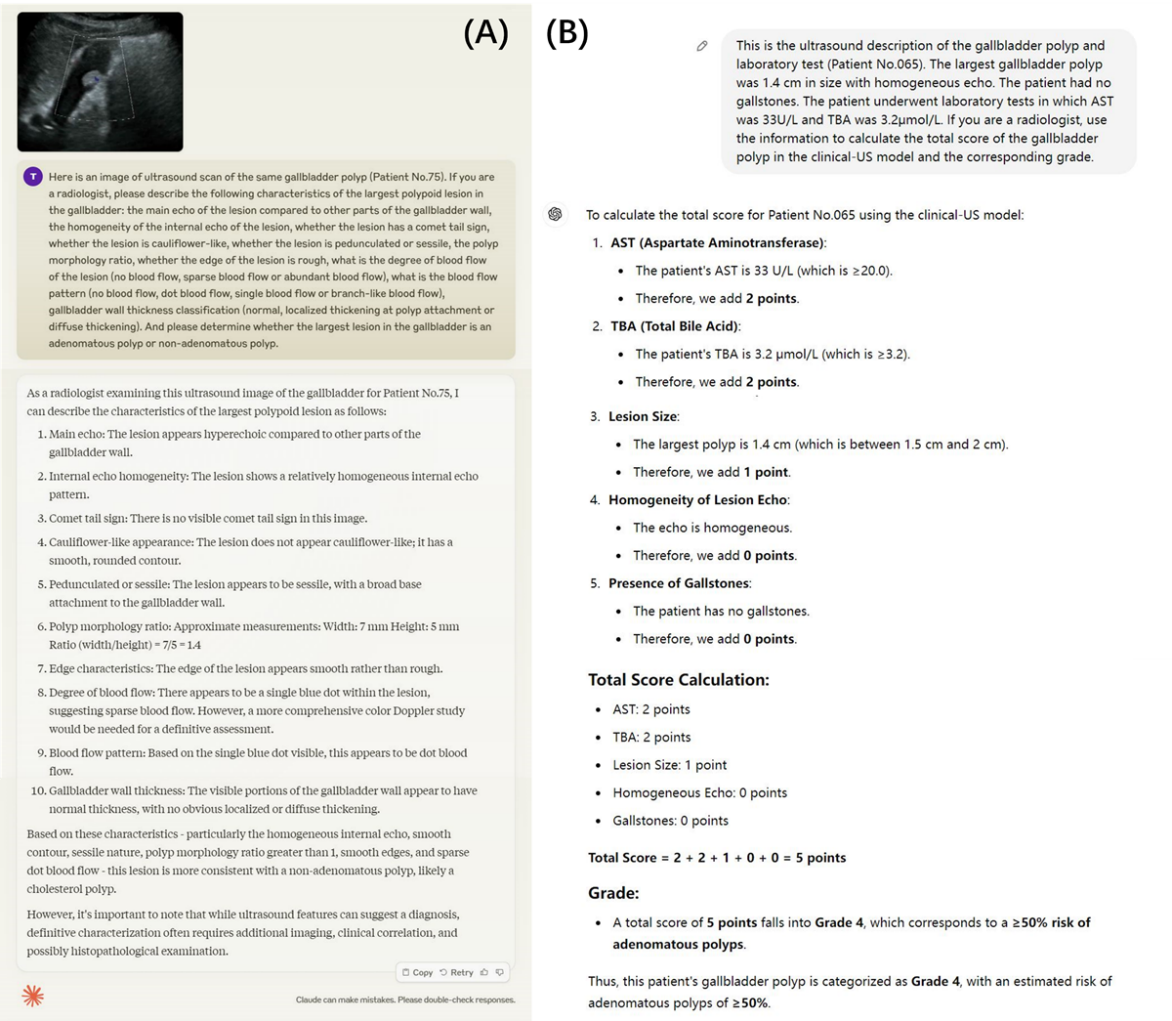
Examples of strategy Claude-image (A) and strategy GPT-model (B). AST: aspartate aminotransferase; TBA: total bile acid.

The diagnostic performance of all diagnostic strategies is presented in Table 4. Figure 4A and B show representative cases from strategy Claude-image and strategy GPT-model, respectively.

The sensitivity of the guideline’s diagnostic strategy was 1.00, indicating that no gallbladder adenomas were missed, but surgery was recommended for all nonneoplastic polyps ≥1.0 cm (UNRR=100%). In the strategies LLMs-image and LLMs-text, the sensitivity of GPT-image, Claude-image, GPT-text, and Claude-text was 0.27, 0.33, 0.56, and 0.65, respectively, which were significantly lower than the sensitivity of the guideline (all *P*<.001), indicating more gallbladder adenomas were missed.

In the strategy readers or LLMs-model, when cholecystectomy was recommended for gallbladder polyps ≥grade 2, the sensitivity of ChatGPT-4o, Claude 3.5 Sonnet, and radiologists were 0.94, 0.98, and 0.98, respectively, showing no significant difference from the sensitivity of 1.00 achieved by the guideline (all *P*<.05). However, the accuracy of ChatGPT-4o, Claude 3.5 Sonnet, and radiologists was significantly higher than that of the guideline (0.35, 0.34, and 0.34 vs 0.22, all *P*<.01), and the UNRR was significantly lower than that of the guideline (82%, 83%, and 83% vs 100%, all *P*<.01). In addition, there were no significant differences observed between GPT-model, Claude-model and readers-model in terms of sensitivity, specificity, PPV, NPV, accuracy or UNRR across all grades (all *P*>.05). Similar results were observed in the external test set (Table S7 in Multimedia Appendix 1).

**Table 4:** Diagnostic performance of all diagnostic strategies. Data in ranges are 95% CIs. Except where indicated, *P* values are for comparison with the guideline that recommends cholecystectomy for polyps ≥1.0 cm.

Diagnostic strategies				Sensitivity		Specificity		PPV^a^		NPV^b^		Accuracy		UNRR^c,d^
**Polyp size ≥1.0 cm**														175/175 (100)
				1.00		0.00		0.22		—^e^		0.22		
				0.93 to 1.00		0.00 to 0.02		0.16 to 0.28		—		0.16 to 0.28		
**GPT-image**														54/175 (31)
				0.27		0.69		0.19		0.78		0.60		
				0.15 to 0.42		0.62 to 0.76		0.11 to 0.31		0.70 to 0.84		0.53 to 0.67		
	*P* value			<.01		<.01		.84		—		<.01		<.001
**Claude-image**														80/175 (46)
				0.33		0.54		0.17		0.75		0.50		
				0.20 to 0.48		0.47 to 0.62		0.10 to 0.26		0.66 to 0.82		0.43 to 0.57		
	*P* value			<.01		<.01		.40		—		<.01		<.001
**GPT-text**														108/175 (62)
				0.56		0.38		0.20		0.76		0.42		
				0.41 to 0.71		0.31 to 0.46		0.14 to 0.28		0.66 to 0.85		0.36 to 0.49		
	*P* value			<.01		<.01		.83		—		<.01		<.001
**Claude-text**														127/175 (73)
				0.65		0.27		0.20		0.74		0.35		
				0.50 to 0.78		0.21 to 0.35		0.14 to 0.27		0.62 to 0.84		0.29 to 0.42		
	*P* value			<.01		<.01		.75		—		<.01		<.001
**GPT-model**														
	≥**Grade 2**													143/175 (82)
			0.94		0.18		0.24		0.91		0.35			
			0.83 to 0.99		0.13 to 0.25		0.18 to 0.31		0.77 to 0.98		0.28 to 0.41			
		*P* value	.24		<.01		.64		—		<.01		<.001	
		*P* value^f^	.61		.78		>.99		.72		>.99		.78	
	≥**Grade 3**													84/175 (48)
			0.75		0.52		0.30		0.88		0.57			
			0.60 to 0.86		0.44 to 0.60		0.22 to 0.39		0.81 to 0.94		0.50 to 0.64			
		*P* value	.01		<.01		.11		—		<.01		<.001	
		*P* value^f^	>.99		.45		.93		>.99		.57		.45	
	**=Grade 4**													5/175 (3)
			0.15		0.97		0.58		0.81		0.79			
			0.06 to 0.28		0.94 to 0.99		0.28 to 0.85		0.75 to 0.86		0.74 to 0.85			
		*P* value	<.01		<.01		.01		—		<.01		<.001	
		*P* value^f^	>.99		.72		.90		>.99		.91		.72	
**Claude-model**														
	≥**Grade 2**													146/175 (83)
			0.98		0.17		0.24		0.97		0.34			
			0.89 to 1.00		0.11 to 0.23		0.19 to 0.31		0.83 to 1.00		0.28 to 0.41			
		*P* value	>.99		<.01		.57		—		<.01		<.001	
		*P* value^f^	>.99		>.99		>.99		>.99		>.99		>.99	
	≥**Grade 3**													93/175 (53)
			0.73		0.47		0.27		0.86		0.53			
			0.58 to 0.85		0.39 to 0.55		0.20 to 0.36		0.78 to 0.93		0.46 to 0.59			
		*P* value	<.01		<.01		.27		—		<.01		<.001	
		*P* value^f^	.81		>.99		.92		.85		.85		>.99	
	**=Grade 4**													5/175 (3)
			0.15		0.97		0.58		0.81		0.79			
			0.06 to 0.28		0.94 to 0.99		0.28 to 0.85		0.75 to 0.86		0.74 to 0.85			
		*P* value	<.01		<.01		.01		—		<.01		<.001	
		*P* value^f^	>.99		.72		.90		>.99		.91		.72	
**Readers-model**														
	≥**Grade 2**													146/175 (83)
			0.98		0.17		0.24		0.97		0.34			
			0.89 to 1.00		0.11 to 0.23		0.19 to 0.31		0.83 to 1.00		0.28 to 0.41			
		*P* value	.99		<.01		.57		—		<.01		<.001	
	≥**Grade 3**													92/175 (53)
			0.77		0.47		0.29		0.88		0.54			
			0.63 to 0.88		0.40 to 0.55		0.21 to 0.37		0.80 to 0.94		0.47 to 0.61			
		*P* value	.01		<.01		.17		—		<.01		<.001	
	**=Grade 4**													3/175 (2)
			0.15		0.98		0.70		0.81		0.80			
			0.06 to 0.28		0.95 to 1.00		0.35 to 0.93		0.75 to 0.86		0.74 to 0.85			
		*P* value	<.01		<.01		<.01		—		<.01		<.001	

^a^PPV: positive predictive value.

^b^NPV: negative predictive value.

^c^UNRR: unnecessary resection rate of nonneoplastic polyps.

^d^n/N (%).

^e^Not applicable.

^f^*P* values are for comparison with the same grade in the strategy readers-model.

### Interpretability of the Diagnosis in Strategy LLMs-Text

The results of univariate analysis based on the diagnosis of GPT-text and Claude-text are shown in Table S8 in Multimedia Appendix 1. Both ChatGPT-4o and Claude 3.5 Sonnet considered larger size, hypoechogenicity, heterogeneous echogenicity, cauliflower shape, sessile base, and rough edge as significant factors for adenomatous polyps. Additionally, ChatGPT-4o identified higher PMR as a diagnostic indicator for adenomatous polyps. Furthermore, Claude 3.5 Sonnet also associated sparse and dot-like blood flow in color Doppler flow imaging with adenomatous polyps. Both LLMs diagnosed all lesions with abundant blood flow (6 cases) as adenomas, which was identified as a risk feature of neoplastic gallbladder polyps by a previous study, while this complete separation led to the extreme or infinite CIs.

### Error Analysis in Strategy LLMs-Text

In the strategy LLMs-text, 129 cases were misdiagnosed by ChatGPT-4o, while 94 cases were correctly diagnosed. As shown in Table S9 in Multimedia Appendix 1, lesions with the following characteristics were more likely to be misdiagnosed by ChatGPT-4o: hypoechoic appearance (23% vs 11%, *P*=.047), heterogeneous echotexture (52% vs 22%, *P*<.001), cauliflower-like morphology (24% vs 13%, *P*=.04), and rough edges (56% vs 29%, *P*<.001).

For Claude 3.5 Sonnet, 144 cases were misdiagnosed by ChatGPT-4o, while 79 cases were correctly diagnosed. Table S10 in Multimedia Appendix 1 demonstrates that Claude 3.5 Sonnet was prone to misdiagnosis in lesions with these features: larger size (1.30 cm vs 1.10 cm, *P*<.001), hypoechoic appearance (21% vs 13%, *P*=.03), heterogeneous echotexture (49% vs 23%, *P*<.001), cauliflower-like morphology (24% vs 11%, *P*=.03), and rough edges (52% vs 30%, *P*=.002).

## Discussion

### Principal Findings

This study evaluated the feasibility of LLMs for the differential diagnosis of gallbladder benign polyps. Our principal findings indicate that the diagnostic strategy profoundly influences LLM performance. In the strategy LLMs-image, LLMs exhibited only slight-to-moderate intrareader agreement and poor interreader consistency compared to radiologists. As the ultrasonic features associated with gallbladder polyps represent common characteristics shared by focal lesions, the finding that current LLMs have limited capabilities in recognizing these features suggests this limitation may have broad applicability. For diagnosis, the diagnostic performance of LLMs-image and the strategy LLMs-text showed significantly lower sensitivity than clinical guidelines, leading to more missed adenomas. In contrast, the strategy LLMs-model, which used text descriptions within a scoring system, demonstrated high consistency and diagnostic performance comparable to radiologists using the same model, while significantly reducing unnecessary cholecystectomies. Notably, the performance between two general-purpose LLMs, ChatGPT-4o and Claude 3.5 Sonnet, was comparable across all strategies, suggesting these findings may generalize to other general-purpose LLMs.

### Comparison With Prior Work

Our results on the poor performance of general-purpose LLMs in direct image interpretation align with a growing body of evidence across various medical fields. Studies using dermoscopic images for melanoma diagnosis, orthopedic residency examination images, and musculoskeletal radiology images consistently report not only unsatisfactory diagnostic accuracy (as low as 3% to 36%) but also highlight that LLM performance is inferior to that of human specialists [34-37]. Critically, some studies report that LLMs could harm patient care by recommending unnecessary invasive procedures, such as biopsies, at a significantly higher rate than radiologists [38]. This collective evidence confirms that current general-purpose LLMs remain inadequate for reliable medical image-based diagnosis.

It is important to note that LLMs receiving specialized training in medical images have demonstrated more competent performance in specific domains, such as dermatology (SkinGPT-4) and diabetes management (DeepDR-LLM) [39,40]. Universal systems such as ChatCAD+ also show promise across multiple image types [41]. However, such specialized models are often confined to a single disease area or a limited set of tasks, and they may underperform advanced general LLMs such as GPT-4 in broader medical question-answering [42]. Other general medical LLMs capable of addressing multiple conditions, such as MedFound, do not yet support image input [43]. Given the critical needs for accessibility, generalizability, and image input capabilities in real-world clinical settings, we selected leading general-purpose LLMs (ChatGPT-4o and Claude 3.5 Sonnet) as the subjects of this study. Our findings contribute to the understanding of their current capabilities and limitations.

Regarding the diagnostic performance of text-based strategies, our findings must be viewed within the context of a highly variable literature. Previous studies report widely varying accuracy (40%-91.4%) for categorical diagnoses using text-only strategies [27,44,45]. When focusing specifically on the interpretation of radiological findings, reported diagnostic accuracy for LLMs ranges from 25% to 73% [26,46-48]. While some studies, such as one on real-world radiology reports of brain tumors, found ChatGPT-4’s accuracy (73%) to be comparable with radiologists [48], the absence of standardized methods and reporting metrics in LLM studies introduces significant bias risks and hinders cross-study comparisons. In our study, the text-based strategy demonstrated superior diagnostic performance to the image-based approach, which aligns with the consensus and the reported performance levels. Nevertheless, given gallbladder adenomas’ high potential for malignancy, the moderate sensitivity achieved by the text-based strategy in our study remains a critical limitation for clinical application, as higher sensitivity is paramount to avoid missed diagnoses.

The LLMs performed suboptimally in the text-based diagnostic strategy, even though the interpretability analysis of the LLMs-text approach indicated that the high-risk features of adenomas identified by the LLMs are supported by previous literature [29,49-51]. In the error analysis, we observed that LLMs showed subpar performance in diagnosing lesions with these same features, such as heterogeneous echotexture and rough edges. Essentially, this finding reflects a long-standing fundamental challenge in the imaging diagnosis of gallbladder polyps: there is significant overlap in the sonographic features between high-grade dysplastic adenomas and early-stage gallbladder cancer. Trained on broader existing literature about medical imaging, the LLMs correctly learned the strong association between these features and common malignancy risk (eg, gallbladder cancer). As indicated by the extreme odds ratio in the interpretability analysis, both LLMs assigned excessive weight to abundant blood flow. Although previous studies confirm it is indeed a feature associated with neoplastic polyps [52], the models’ tendency to treat it as an absolute diagnostic rule—rather than a probabilistic indicator—reveals their limitation in performing the task of fine-grained diagnosis, a reflection of the inherent difficulty of the task itself. However, when the task was confined to the relatively idealized binary framework of “differentiating adenomatous from non-neoplastic polyps,” the model’s heightened sensitivity to these high-risk features created a conflict with the task objective. This may not represent a “weakness” in the model, but rather an objective reflection of complex clinical reality—the degree of dysplasia in lesions exists on a spectrum, and such ambiguity in imaging is inherent before the intervention of the pathological “gold standard.” Meanwhile, the LLMs also demonstrated valuable capabilities. For instance, ChatGPT-4o correctly recognized a novel morphological index (PMR) from an unpublished study as useful for diagnosis, and Claude 3.5 Sonnet identified sparse and dot blood flow as a relevant feature, which aligns with established research [29,49]. This demonstrates their potential in tasks requiring strong information retrieval and logical reasoning.

Conversely, our study reinforces that LLMs exhibit excellent performance when working with structured text data from scoring systems. Their high accuracy and consistency in applying such systems are supported by prior research in Liver Imaging Reporting and Data System and Thyroid Imaging Reporting and Data System classification [9,53]. In addition, our findings clarify the role of general-purpose LLMs. In the LLMs-model strategy, the LLM acts as a reliable executor of clinical rules, not a simple calculator. It first understands text to find key features, then applies the scoring rule. The performance of our LLMs-model strategy, which was comparable to radiologists, suggests that leveraging LLMs to execute standardized clinical rules may be their most reliable and immediate clinical application.

### Strengths and Limitations

A key strength of this study is the comprehensive evaluation of LLMs across three distinct diagnostic strategies, providing a clear understanding of their appropriate clinical roles. We also provided an in-depth analysis of their reasoning, identifying both capabilities and weaknesses.

This study has several limitations. First, the task of differentiating benign gallbladder polyps is inherently challenging, even for radiologists, which may have contributed to the LLMs’ suboptimal performance. Second, the evaluation was conducted on a single disease entity; assessing broader clinical applicability requires more diverse and complex cases. Third, we used in-context learning with only typical cases. While this approach simulates a realistic usage scenario and is highly accessible, it is inherently less stable than fine-tuning and may not capture the full spectrum of clinical presentations. Finally, the retrospective design may introduce potential selection biases.

### Future Directions

Based on our findings, future research should focus on several key areas. First, the development and evaluation of more versatile and medically tuned multimodal LLMs are crucial. Second, the performance of LLMs is heavily dependent on the quality of the underlying scoring system; therefore, integrating them with more robust and validated clinical models could maximize their diagnostic utility. Finally, standardizing evaluation methods and reporting metrics across LLM studies is urgently needed to enable meaningful comparisons. Given their strong performance in information retrieval and logical reasoning, future work should also explore the role of LLMs in medical education [54,55] and self-management counseling for patients with chronic conditions [56].

### Conclusions

In conclusion, current general-purpose LLMs have poor reproducibility and diagnostic performance in image-based diagnosis of gallbladder polyps, limiting their direct clinical application. However, they demonstrate significant potential when used in a text-based strategy that uses a clinical scoring system, achieving performances comparable to radiologists. This model-based approach currently represents the most appropriate diagnostic strategy for LLMs in this domain.

## Appendix

Multimedia Appendix 1 Study protocols, prompt templates, statistical details, and extended results.

Multimedia Appendix 2 Example No.1. An example of dot and sparse blood flow.

Multimedia Appendix 3 Example No.2. An example of single and sparse blood flow.

Multimedia Appendix 4 Example No.3. An example of branch-like and abundant blood flow.

Multimedia Appendix 5 Example No.4. An example of a pedunculated polyp.

Multimedia Appendix 6 Example No.5. An example of a sessile polyp.

## Abbreviations

CLAIM: Checklist for Artificial Intelligence in Medical Imaging
LLM: large language model
NPV: negative predictive value
PASS: power analysis and sample size
PMR: polyp morphology ratio
PPV: positive predictive value
UNRR: unnecessary resection rate of nonneoplastic polyps

## Data Availability

The original ultrasound images and clinical records analyzed during this study are not publicly available due to patient confidentiality and privacy protection regulations. However, deidentified data underlying the reported results can be made available from the corresponding author upon reasonable request. Requests should include a detailed research proposal outlining the intended use of the data and must be approved by the institutional ethics committee that oversaw the original study.
