# Supplementary material for: Evaluating Multiple Input Strategies of Large Language Models for Gallbladder Polyps on Ultrasound: Comparative Study
Source: JMIR Med Inform. 2025 Dec 23;13:e71178. doi: 10.2196/71178 (PMC12777648; doi:10.2196/71178)
Supplement: Multimedia Appendix 1 [file medinform_v13i1e71178_app1.docx]

**Appendix 1**

**Definitions of Ultrasound Features**

The size of the gallbladder polyp and the thickness of the gallbladder wall are measured in centimeters, with one decimal place retained. The echo of the polyp is compared to the echo of the gallbladder wall. The base of the polyp is defined as where the lesion joined the wall of the gallbladder. If the base of the lesion is the widest of the entire lesion, it is sessile; otherwise, it is pedunculated (Multimedia Appendix 5&6). The polyp morphology ratio (PMR) is calculated as the width of the polyp base / the width of the widest part parallel to the gallbladder wall. The blood flow pattern of color Doppler flow imaging (CDFI) is divided into no flow, dot, single and branch-like [29]. The thickness of gallbladder wall ≥ 3 mm is considered as thickening [30].

**Ultrasound Examination Protocol**

Patients were required to fast for at least 8 hours prior to the examination. The scans were performed with the patient in the supine or left lateral decubitus position. Grayscale ultrasound and color Doppler flow imaging (CDFI) were used to examine the gallbladder. Images were magnified, and parameters such as depth, focus, gain, and velocity scale were adjusted to optimize visualization of the target lesion. Static images were captured in the plane that provided the clearest view of the lesion's maximum cross-section, including its base.

In this study, the following ultrasound systems were used for gallbladder polyp imaging:Acuson Sequoia 512 (Siemens Medical Solutions, Mountain View, California), Aplio XV (Toshiba Medical Systems, Tokyo, Japan), Aixplorer X (Supersonic Imaging, Aix-en-Provence, France), Aplio i900 (Canon, Tokyo, Japan), All systems were equipped with abdominal convex array probes (1–6 MHz). In a limited number of cases, linear array probes (5–10 MHz) were additionally employed for image acquisition.

**Prompts Template in Diagnostic Strategies for LLMs**

**Strategy LLM-Image**

Here are two images of ultrasound scan of the same gallbladder polyp (Patient No.01). If you are a radiologist, please describe the following characteristics of the largest polypoid lesion in the gallbladder: the main echo of the lesion compared to other parts of the gallbladder wall, the homogeneity of the internal echo of the lesion, whether the lesion has a comet tail sign, whether the lesion is cauliflower-like, whether the lesion is pedunculated or sessile, the polyp morphology ratio, whether the edge of the lesion is rough, what is the degree of blood flow of the lesion (no blood flow, sparse blood flow or abundant blood flow), what is the blood flow pattern (no blood flow, dot blood flow, single blood flow or branch-like blood flow), gallbladder wall thickness classification (normal, localized thickening at polyp attachment or diffuse thickening). And please determine whether the largest lesion in the gallbladder is an adenomatous polyp or non-neoplastic polyp.

**Strategy LLM-Text**

This is a text description of the ultrasonographic manifestations of gallbladder polyps (Patient No.01). This patient had single gallbladder polyp/multiple gallbladder polyps, and the largest of which was 1.0cm in size. The largest lesion is hypoechoic/isoechoic/hyperechoic, and the internal echo is homogeneous/heterogeneous, without/with the comet-tail artifact. The shape of the lesion was cauliflower/non-cauliflower. The polyp was pedunculated/sessile, and the polyp morphology ratio was 0.65. The edge of the lesion was rough/smooth. Color Doppler flow imaging showed that the lesion had no blood flow/sparse blood flow/abundant blood flow, and the blood flow pattern was no blood flow/dot/single/branch-like. The thickness of the gallbladder wall was 0.3 cm, and the gallbladder wall thickness classification was normal/localized thickening at polyp attachment/diffuse thickening. The patient had (no) gallstones. If you are a radiologist, please determine whether the largest polyp in the gallbladder is an adenomatous polyp or non-neoplastic polyp.

**Strategy LLM-Model**

This is the ultrasound description of the gallbladder polyp and laboratory test (Patient No.01). The largest gallbladder polyp was 1.0cm in size with homogeneous/heterogeneous echo. The patient had (no) gallstones. The patient underwent laboratory tests in which AST was 20U/L and TBA was 3.2μmol/L. If you are a radiologist, use the information to calculate the total score of the gallbladder polyp in the clinical-US model and the corresponding grade.

**Prompts in In-context Learning for LLMs**

**In-context Learning in Blood Flow Levels and Patterns**

Are you able to recognize the features of gallbladder polyps in gallbladder ultrasound images? Here are some representative ultrasound images that characterize gallbladder polyps.

1. Here is the first example. In this image (Example No.1), the blood flow pattern of the gallbladder polyp is dot and the blood flow degree is sparse.
2. Here is the second example. In this image (Example No.2), the blood flow pattern of the gallbladder polyp is single and the blood flow degree is sparse.
3. Here is the third example. In this image (Example No.3), the blood flow pattern of the gallbladder polyp is branch-like and the blood flow degree is abundant.

**In-context Learning for Polyp Morphology Ratio, Sessile/Pedunculated Base Definitions**

The base of the polyp is defined as the part where the lesion joins the gallbladder wall. If the width of the lesion base is the widest part of the entire lesion, it is sessile, otherwise it is pedunculated. The polyp morphology ratio is calculated as the width of the polyp base/the width of the widest part parallel to the gallbladder wall. The range of the polyp morphology ratio is 0 to 1. The polyp morphology ratio of a sessile polyp is 1.00, and the ratio of a pedunculated polyp is less than 1.00.

1. Here is the 4th example. In this image (Example No.4), the polyp is pedunculated because the base of the polyp is not the widest part of the polyp. Its polyp morphology ratio is 0.40.
2. Here is the 5th example. In this image (Example No.5), the polyp is sessile because the widest part of the polyp is the base. Its polyp morphology ratio is 1.00.

**In-context Learning for Scoring System**

Here is a diagnostic model of gallbladder polyps based on clinical and ultrasonic features to distinguish adenomatous polyps from non-adenomatous polyps. This model has five indicators, including serum concentration of aspartate aminotransferase (AST), serum concentration of total bile acid (TBA), lesion size, homogeneity of lesion echo, gallstones. If the AST is ≥20.0, add 2 points, otherwise add 0 points. If TBA≥3.2, add 2 points, otherwise add 0 points. Add 0 points if the echo is homogeneous, subtract 1 point if the echo is heterogeneous. Subtract 4 points if combined with gallstones, otherwise add 0 points. If 1cm≤ size < 1.5cm, add 0 points; If 1.5cm≤ size < 2cm, add 1 point; If size≥2cm, add 3 points. The score of the five indicators is added together to obtain the total score. A total score of -5 to -1 is grade 1, 0 to 1 is grade 2, 2 to 4 is grade 3, and 5 to 7 is grade 4. The estimated risk of adenomatous polyps in grade 1, 2, 3, and 4 is 0%-10%, 10%-20%, 20%-50%, and ≥50%, respectively.

**Detailed Sample Size Calculations**

1. For Intra-reader Agreement Analysis

Objective: To ensure a precise estimate of the intra-reader agreement (ICC) for each Large Language Model (LLM) strategy, based on the variability observed in our preliminary data.

Software: PASS 2025, using the "Confidence Intervals for Intraclass Correlation" module.

Result: The calculation indicated that a sample size of N = 67 subjects was required. This was rounded up to a target of N = 70 subjects for the study.

2. For Comparison of LLM-Image vs. LLM-Text Diagnostic Strategies

Objective: To determine the sample size required to detect a statistically significant difference in reader agreement between the LLMs-Image and LLMs-Text strategies, with a power of 80% and a two-sided alpha of 0.05.

Software: PASS 2025, using the "Tests for Two Correlated Proportions (McNemar Test)" module, based on the preliminary agreement results from 20 cases.

Result: The calculation indicated that a total of N = 234 evaluable cases would be required. Considering practical constraints and the primary focus on model validation, the sample size allocated for the internal reader agreement analysis was set at N = 70 subjects.

3. For External Validation of the LLMs-Model Strategy

Objective: To determine the sample size for the external validation cohort to estimate the model's sensitivity with a pre-specified precision.

Software: PASS 2025, using the "Confidence Intervals for One-Sample Sensitivity" module.

Result: The calculation indicated that a total sample size of N = 97 subjects was required. This was rounded up to a target of N = 100 subjects for the external test set.

Table S1. Checklist for artificial intelligence in medical imaging

| Section/Topic | No. | Item | √ (N/A) |
| --- | --- | --- | --- |
| **TITLE/ABSTRACT** |  |  |  |
|  | 1 | Identification as a study of AI methodology, specifying the category of technology used (eg, deep learning) | √ |
| **ABSTRACT** | 2 | Summary of study design, methods, results, and conclusions | √ |
| **INTRODUCTION** | 3 | Scientific and/or clinical background, including the intended use and role of the AI approach | √ |
|  | 4 | Study aims, objectives, and hypotheses | √ |
| **METHODS** |  |  |  |
| **Study Design** | 5 | Prospective or retrospective study | √ |
|  | 6 | Study goal | √ |
| **Data** | 7 | Data sources | √ |
|  | 8 | Inclusion and exclusion criteria | √ |
|  | 9 | Data preprocessing | √ |
|  | 10 | Selection of data subsets | √ |
|  | 11 | De-identification methods | √ |
|  | 12 | How missing data were handled | N/A (No missing data) |
|  | 13 | Image acquisition protocol | √ |
| **Reference Standard** | 14 | Definition of method(s) used to obtain reference standard | √ |
|  | 15 | Rationale for choosing the reference standard | √ |
|  | 16 | Source of reference standard annotations | N/A (Reference standard was histopathological results, not image annotation) |
|  | 17 | Annotation of test set | N/A (Same as above) |
|  | 18 | Measures of inter- and intraater variability of features described by the annotators | N/A (Assessed inter-reader agreement, not annotator variability for the reference standard) |
| **Data Partitions** | 19 | How data were assigned to partitions | N/A (No data partitioning was performed) |
|  | 20 | Level at which partitions are disjoint | N/A (Same as above) |
| **Testing Data** | 21 | Intended sample size | √ |
| **Model** | 22 | Detailed description of model | √ |
|  | 23 | Software libraries, frameworks, and packages | √ |
|  | 24 | Initialization of model parameters | √ |
| **Training** | 25 | Details of training approach | N/A (No model training or fine-tuning was performed) |
|  | 26 | Method of selecting the final model | N/A (Same as above) |
|  | 27 | Ensembling techniques | N/A (No model ensembling was used) |
| **Evaluation** | 28 | Metrics of model performance | √ |
|  | 29 | Statistical measures of significance and uncertainty | √ |
|  | 30 | Robustness or sensitivity analysis | N/A (Not feasible due to continuous updates and unavailability of the original LLM versions) |
|  | 31 | Methods for explainability or interpretability | √ |
|  | 32 | Evaluation on internal data | √ |
|  | 33 | Testing on external data | √ |
|  | 34 | Clinical trial registration | N/A |
| **RESULTS** |  |  |  |
| **Data** | 35 | Numbers of patients or examinations included and excluded | √ |
|  | 36 | Demographic and clinical characteristics of cases in each partition | √ |
| **Model Performance** | 37 | Performance metrics and measures of statistical uncertainty | √ |
|  | 38 | Estimates of diagnostic performance and their precision | √ |
|  | 39 | Failure analysis of incorrectly classified cases | √ |
| **DISCUSSION** | 40 | Study limitations | √ |
|  | 41 | Implications for practice, including intended use and/or clinical role | √ |
| **OTHER INFORMATION** | 42 | Provide a reference to the full study protocol or to additional technical details | √ |
|  | 43 | Statement about the availability of software, trained model, and/or data | √ |
|  | 44 | Sources of funding and other support; role of funders | √ |

Table S2. Reproducibility Checklist

| No. | Item | Status & Location | Notes & Justifications |
| --- | --- | --- | --- |
| 1 | **Complete Prompt Templates** | Provided **Appendix 1** | All exact prompts and in-context learning examples for every diagnostic strategy (LLMs-Image, LLMs-Text, LLMs-Model) are fully detailed. |
| 2 | **Model Specifications** | Provided **Methods Section** | The specific model versions (e.g., ChatGPT-4o, Claude 3.5 Sonnet) and the time period of evaluation (July - September 2024) are clearly stated. |
| 3 | **Input Data Specifications** | Provided **Methods Section** | Detailed descriptions of image pre-processing (cropping, resolution) and text report structuring are included. |
| 4 | **Diagnostic Scoring System Rules** | Provided **Table S3** | The full multi-level scoring system, including all variables, scores, and grade thresholds used in the 'Strategy LLMs-Model', is provided. |
| 5 | **Example LLM Outputs** | Provided **Figure 4** | Representative examples of the LLM interaction workflow, from input prompt to output, are illustrated in the main figure. |
| 6 | **Reporting Guideline Checklist** | Provided **Table S1** | A completed CLAIM (Checklist for Artificial Intelligence in Medical Imaging) checklist is provided to ensure comprehensive reporting. |
| 7 | **Original Patient Data** | Not Applicable (Ethical Restrictions) | Original ultrasound images and medical records cannot be shared publicly due to patient privacy and confidentiality protections. |
| 8 | **Custom Code & API Parameters** | Not Applicable (Platform Limitation) | The study was conducted via standard web interfaces without custom code. Specific non-default API parameters were not used. |

Table S3. Clinical-US model scoring system

| Risk factor | Categories | Points |
| --- | --- | --- |
| Polyp size | 1cm ≤ d＜1.5cm | 0 |
|  | 1.5cm ≤ d＜2cm | 1 |
|  | ≥ 2cm | 3 |
| Echo uniformity | Yes | 0 |
|  | No | -1 |
| Gallstones | No | 0 |
|  | Yes | -4 |
| AST ≥ 20.0U/L | No | 0 |
|  | Yes | 2 |
| TBA ≥ 3.2μmol/L | No | 0 |
|  | Yes | 2 |

d, diameter; AST, aspartate aminotransferase; TBA, total bile acid.

The total points range for different grades is as follows: Grade 1 (-5 to -1), Grade 2 (0 to 1), Grade 3 (2 to 4), Grade 4 (5 to 7). And the range of estimated risk of Grade 1-4 is 0%-10%, 10%-20%, 25%-50%, and ≥50%, respectively.

Table S4. Intra-reader agreement coefficients in strategy LLM – Model in external test cohort

|  | ChatGPT-4o | Claude 3.5 Sonnet |
| --- | --- | --- |
| Total score^a^ | 1.00 (-)^c^ | 1.00 (-)^c^ |
| Grade^b^ | 0.94 (0.89, 0.97) | 0.98 (0.96, 0.99) |

Note.—Data in parentheses are 95% CIs.

^a^The coefficient of agreement analysis is ICC.

^b^The coefficient of agreement analysis is Kendall's W coefficient.

^c^Confidence intervals cannot be calculated due to perfect consistency of three rounds output in each case.

Table S5. Inter-reader agreement coefficients in human readers and LLMs

|  | Reader 1  vs.  Reader 2 | Reader 1  vs.  GPT | Reader 1  vs.  Claude | Reader 2  vs.  GPT | Reader 2  vs.  Claude | Readers  vs.  GPT | Readers  vs.  Claude | GPT  vs.  Claude |
| --- | --- | --- | --- | --- | --- | --- | --- | --- |
| Polyp morphology ratio^a^ | 0.65  (0.57, 0.72) | 0.10  (-0.03, 0.23) | 0.07  (-0.06, 0.20) | 0.04  (-0.09, 0.17) | 0.06  (-0.07, 0.19) | 0.08  (-0.06, 0.21) | 0.08  (-0.06, 0.21) | 0.00  (-0.13, 0.13) |
| *P* Value | - | ＜.001 | ＜.001 | ＜.001 | ＜.001 | ＜.001 | ＜.001 | ＜.001 |
| Echo level^b^ | 0.85  (0.80, 0.90) | 0.15  (0.02, 0.28) | 0.05  (-0.03, 0.14) | 0.13  (0.01, 0.25) | 0.05  (-0.02, 0.11) | 0.13  (0.01, 0.24) | 0.04  (-0.02, 0.11) | -0.02  (-0.13, 0.09) |
| *P* Value | - | ＜.001 | ＜.001 | ＜.001 | ＜.001 | ＜.001 | ＜.001 | ＜.001 |
| Echo uniformity | 0.76  (0.67, 0.85) | 0.01  (-0.09, 0.12) | 0.09  (-0.02, 0.20) | 0.02  (-0.10, 0.13) | 0.06  (-0.06, 0.18) | 0.00  (-0.11, 0.11) | 0.07  (-0.05, 0.18) | 0.02  (-0.11, 0.16) |
| *P* Value | - | ＜.001 | ＜.001 | ＜.001 | ＜.001 | ＜.001 | ＜.001 | ＜.001 |
| Comet tail sign | NA | NA | NA | -0.01  (-0.01, 0.00) | -0.02  (-0.04, 0.00) | -0.01  (-0.01, 0.00) | -0.02  (-0.04, 0.00) | -0.01  (-0.02, 0.01) |
| *P* Value | - | - | - | - | - | - | - | - |
| Cauliflower shape | 0.57  (0.43, 0.72) | -0.01  (-0.03, 0.01) | -0.05  (-0.16, 0.07) | -0.01  (-0.03, 0.01) | 0.00  (-0.13, 0.13) | -0.01  (-0.03, 0.01) | 0.01  (-0.12, 0.14) | -0.01  (-0.03, 0.01) |
| *P* Value |  | ＜.001 | ＜.001 | ＜.001 | ＜.001 | ＜.001 | ＜.001 | ＜.001 |
| Pedunculated/sessile | 0.86  (0.79, 0.94) | 0.04  (-0.09, 0.17) | -0.11  (-0.21, -0.01) | 0.01  (-0.12, 0.14) | -0.10  (-0.19, 0.00) | 0.00  (-0.12, 0.13) | -0.09  (-0.19, 0.00) | -0.01  (-0.12, 0.10) |
| *P* Value | - | ＜.001 | ＜.001 | ＜.001 | ＜.001 | ＜.001 | ＜.001 | ＜.001 |
| Edge | 0.82  (0.75, 0.89) | 0.06  (0.01, 0.11) | 0.05  (-0.04, 0.15) | 0.07  (0.00, 0.13) | 0.07  (-0.04, 0.18) | 0.06  (0.00, 0.12) | 0.07  (-0.04, 0.18) | 0.02  (-0.09, 0.13) |
| *P* Value | - | ＜.001 | ＜.001 | ＜.001 | ＜.001 | ＜.001 | ＜.001 | ＜.001 |
| Blood flow degree^b^ | 0.99  (0.97, 1.00) | 0.52  (0.40, 0.64) | 0.47  (0.34, 0.60) | 0.52  (0.40, 0.64) | 0.47  (0.34, 0.60) | 0.52  (0.40, 0.64) | 0.47  (0.34, 0.60) | 0.75  (0.68, 0.82) |
| *P* Value | - | ＜.001 | ＜.001 | ＜.001 | ＜.001 | ＜.001 | ＜.001 | ＜.001 |
| Blood flow pattern | 0.92  (0.86, 0.98) | 0.39  (0.35, 0.43) | 0.34  (0.30, 0.38) | 0.40  (0.35, 0.44) | 0.34  (0.31, 0.38) | 0.40  (0.36, 0.44) | 0.34  (0.30, 0.39) | 0.69  (0.60, 0.78) |
| *P* Value | - | ＜.001 | ＜.001 | ＜.001 | ＜.001 | ＜.001 | ＜.001 | ＜.001 |
| Gallbladder wall thickness types | 0.77  (0.63, 0.92) | 0.06  (-0.02, 0.15) | -0.01  (-0.08, 0.06) | 0.05  (-0.05, 0.15) | -0.06  (-0.11, 0.00) | 0.05  (-0.04, 0.14) | -0.02  (-0.10, 0.05) | 0.06  (-0.09, 0.20) |
| *P* Value | - | ＜.001 | ＜.001 | ＜.001 | ＜.001 | ＜.001 | ＜.001 | ＜.001 |

^a^The coefficient of agreement analysis refers to ICC

^b^The coefficient of agreement analysis refers to weighted Kappa.

The other coefficient of agreement analysis refers to Cohen’s Kappa. P values are for comparison with Reader 1 vs. Reader 2.

Table S6. Inter-reader agreement coefficients in strategy Readers/LLM – Model in external test cohort

|  | Readers  vs.  ChatGPT-4o | Readers  vs.  Claude 3.5 Sonnet | ChatGPT-4o  vs.  Claude 3.5 Sonnet |
| --- | --- | --- | --- |
| Total score^a^ | 0.96 (0.94, 0.97) | 0.97 (0.95, 0.98) | 0.98 (0.97, 0.99) |
| Grade^b^ | 0.82 (0.74, 0.88) | 0.93 (0.88, 0.96) | 0.85 (0.77, 0.90) |

Note.—Data in parentheses are 95% CIs.

^a^The coefficient of agreement analysis is ICC.

^b^The coefficient of agreement analysis is weighted Kappa.

Table S7. Diagnostic performance of guideline and strategy Readers/LLM – Model in external test cohort

| Diagnostic strategies | Sensitivity | Specificity | PPV | NPV | Accuracy | UNRR |
| --- | --- | --- | --- | --- | --- | --- |
| Polyp size ≥ 1.0cm | 1.00 | 0.00 | 0.21 | - | 0.21 | 100%  (73/73) |
|  | (0.92, 1.00) | (0.00, 0.04) | (0.14, 0.29) |  | (0.14, 0.29) |  |
| GPT-Model |  |  |  |  |  |  |
| ≥ Grade 2 | 0.92 | 0.16 | 0.23 | 0.88 | 0.33 | 84%  (61/73) |
|  | (0.80, 0.98) | (0.09, 0.24) | (0.15, 0.32) | (0.72, 0.96) | (0.24, 0.43) |  |
| *P* Value | .47 | .01 | .83 | - | .02 | ＜.001 |
| *P* Value^a^ | ＞.99 | ＞.99 | ＞.99 | ＞.99 | ＞.99 | .94 |
| ≥ Grade 3 | 0.72 | 0.49 | 0.28 | 0.86 | 0.55 | 51%  (37/73) |
|  | (0.56, 0.85) | (0.38, 0.59) | (0.18, 0.40) | (0.76, 0.93) | (0.45, 0.65) |  |
| *P* Value | .01 | ＜.01 | .31 | - | ＜.01 | ＜.001 |
| *P* Value^a^ | ＞.99 | .71 | ＞.99 | ＞.99 | .87 | .90 |
| = Grade 4 | 0.12 | 0.96 | 0.50 | 0.79 | 0.77 | 4%  (3/73) |
|  | (0.03, 0.27) | (0.90, 0.99) | (0.21, 0.79) | (0.69, 0.87) | (0.67, 0.85) |  |
| *P* Value | ＜.01 | ＜.01 | .03 | - | ＜.01 | ＜.001 |
| *P* Value^a^ | ＞.99 | .83 | .82 | ＞.99 | ＞.99 | .83 |
| Claude-Model |  |  |  |  |  |  |
| ≥ Grade 2 | 0.96 | 0.15 | 0.23 | 0.93 | 0.32 | 85%  (62/73) |
|  | (0.85, 1.00) | (0.08, 0.23) | (0.15, 0.32) | (0.78, 0.99) | (0.23, 0.42) |  |
| *P* Value | ＞.99 | ＜.01 | .78 | - | ＜.01 | ＜.001 |
| *P* Value^a^ | ＞.99 | ＞.99 | ＞.99 | ＞.99 | ＞.99 | ＞.99 |
| ≥ Grade 3 | 0.70 | 0.45 | 0.25 | 0.85 | 0.51 | 55%  (40/73) |
|  | (0.54, 0.83) | (0.34, 0.55) | (0.16, 0.36) | (0.74, 0.92) | (0.41, 0.61) |  |
| *P* Value | ＜.01 | ＜.01 | .45 | - | ＜.01 | ＜.001 |
| *P* Value^a^ | .81 | ＞.99 | .91 | .69 | .78 | ＞.99 |
| = Grade 4 | 0.12 | 0.96 | 0.50 | 0.79 | 0.77 | 4%  (3/73) |
|  | (0.03, 0.27) | (0.90, 0.99) | (0.21, 0.79) | (0.69, 0.87) | (0.67, 0.85) |  |
| *P* Value | ＜.01 | ＜.01 | .03 | - | ＜.01 | ＜.001 |
| *P* Value^a^ | ＞.99 | .83 | .82 | ＞.99 | ＞.99 | .83 |
| Readers-Model |  |  |  |  |  |  |
| ≥ Grade 2 | 0.96 | 0.15 | 0.23 | 0.93 | 0.32 | 85%  (62/73) |
|  | (0.85, 1.00) | (0.08, 0.23) | (0.15, 0.32) | (0.78, 0.99) | (0.28, 0.42) |  |
| *P* Value | ＞.99 | ＜.01 | .78 | - | ＜.01 | ＜.001 |
| ≥ Grade 3 | 0.72 | 0.46 | 0.26 | 0.86 | 0.52 | 53%  (39/73) |
|  | (0.57, 0.85) | (0.36, 0.56) | (0.17, 0.37) | (0.76, 0.93) | (0.42, 0.62) |  |
| *P* Value | ＜.01 | ＜.01 | .38 | - | ＜.01 | ＜.001 |
| = Grade 4 | 0.12 | 0.98 | 0.66 | 0.79 | 0.78 | 3%  (2/73) |
|  | (0.03, 0.31) | (0.92, 1.00) | (0.30, 0.93) | (0.69, 0.87) | (0.68, 0.86) |  |
| *P* Value | ＜.01 | ＜.01 | ＜.01 | - | ＜.01 | ＜.001 |

Note.—Data in parentheses are 95% CIs. Except where indicated, P values are for comparison with the guideline that recommends cholecystectomy for polyps ≥ 1.0cm. PPV, positive predictive value; NPV, negative predictive value; UNNR, unnecessary resection rate of nonneoplastic polyps

^a^*P* values are for comparison with same grade in strategy Readers-Model.

Table S8. Univariate analysis in strategy LLMs-Text

|  | GPT - Text | | |  | Claude - Text | | |
| --- | --- | --- | --- | --- | --- | --- | --- |
| Variables | OR | 95% CI | *P* Value |  | OR | 95% CI | *P* Value |
| Number of polyps |  |  |  |  |  |  |  |
| multiple |  | Reference |  |  |  | Reference |  |
| single | 1.31 | 0.76-2.26 | 0.33 |  | 1.27 | 0.71-2.30 | 0.42 |
| Size of the largest polyp (cm) | 4.09 | 1.59-10.57 | 0.004* |  | 67.49 | 12.43-366.43 | ＜0.001* |
| Echogenicity |  |  |  |  |  |  |  |
| hypoecho |  | Reference |  |  |  | Reference |  |
| isoecho | 0.25 | 0.10-0.63 | 0.003* |  | 0.19 | 0.06-0.67 | 0.01* |
| hyperecho | 0.29 | 0.11-0.72 | 0.01* |  | 0.13 | 0.04-0.45 | 0.001* |
| Echo uniformity |  |  |  |  |  |  |  |
| yes |  | Reference |  |  |  | Reference |  |
| no | 3.80 | 2.06-6.99 | ＜0.001* |  | 5.36 | 2.55-11.27 | ＜0.001* |
| Comet tail sign | 0.00 | 0.00-∞ | 0.99 |  | 0.00 | 0.00-∞ | 0.99 |
| Cauliflower shape | 2.96 | 1.14-6.52 | 0.01* |  | 5.00 | 1.71-14.63 | 0.003* |
| Base type |  |  |  |  |  |  |  |
| pedunculated |  | Reference |  |  |  | Reference |  |
| sessile | 2.36 | 1.27-4.41 | 0.01* |  | 2.20 | 1.10-4.38 | 0.03* |
| Polyp morphology ratio | 3.01 | 1.24-7.28 | 0.02* |  | 2.23 | 0.87-5.72 | 0.10 |
| Edge |  |  |  |  |  |  |  |
| rough |  | Reference |  |  |  | Reference |  |
| smooth | 0.25 | 0.14-0.45 | ＜0.001* |  | 0.21 | 0.11-0.42 | ＜0.001* |
| Blood flow degree |  |  |  |  |  |  |  |
| none |  | Reference |  |  |  | Reference |  |
| sparse | 1.45 | 0.68-3.07 | 0.34 |  | 5.73 | 1.69-19.43 | 0.01* |
| abundant | 1.17×10^9^ | 0.00-∞ | 0.99 |  | 8.42×10^8^ | 0.00-∞ | 0.99 |
| Blood flow pattern |  |  |  |  |  |  |  |
| none |  | Reference |  |  |  | Reference |  |
| dot | 2.17 | 0.82-5.73 | 0.12 |  | 5.73 | 1.31-25.17 | 0.02* |
| single | 1.30 | 0.42-4.04 | 0.65 |  | 6.77 | 0.87-52.98 | 0.07 |
| branch-like | 2.17 | 0.22-21.28 | 0.51 |  | 8.42×10^8^ | 0.00-∞ | 0.99 |
| Gallbladder wall thickness (cm) | 6.41 | 0.06-637.19 | 0.43 |  | 5.68 | 0.04-813.42 | 0.49 |
| Gallbladder wall thickness types |  |  |  |  |  |  |  |
| normal |  | Reference |  |  |  | Reference |  |
| local thickening of the polyp attachment site | 1.06×10^9^ | 0.00-∞ | 0.99 |  | 6.89×10^8^ | 0.00-∞ | 0.99 |
| diffuse thickening | 0.66 | 0.22-1.95 | 0.45 |  | 1.07 | 0.32-3.53 | 0.92 |
| Gallstones | 1.21 | 0.43-3.41 | 0.72 |  | 2.01 | 0.56-7.24 | 0.29 |

Table S9. Error analysis in strategy LLMs-Text for ChatGPT-4o

|  | Correct cases (n=94) | Error cases (n=129) | *P* Value |
| --- | --- | --- | --- |
| Number of polyps |  |  |  |
| multiple | 54 (60%) | 72 (56%) | 0.587 |
| single | 38 (40%) | 57 (44%) |  |
| Size of the largest polyp (cm) | 1.20 (1.10-1.50) | 1.30 (1.10-1.50) | 0.161 |
| Echogenicity |  |  |  |
| hypoecho | 10 (11%) | 30 (23%) | 0.047* |
| isoecho | 45 (48%) | 56 (43%) |  |
| hyperecho | 39 (42%) | 43 (33%) |  |
| Echo uniformity |  |  |  |
| yes | 73 (78%) | 62 (48%) | ＜0.001* |
| no | 21 (22%) | 67 (52%) |  |
| Comet tail sign | 1 (1%) | 1 (1%) | ＞0.999 |
| Cauliflower shape | 12 (13%) | 31 (24%) | 0.040* |
| Base type |  |  |  |
| pedunculated | 67 (71%) | 87 (67%) | 0.561 |
| sessile | 27 (29%) | 42 (33%) |  |
| Polyp morphology ratio | 0.55 (0.39-1.00) | 0.54 (0.36-1.00) | 0.905 |
| Edge |  |  |  |
| rough | 27 (29%) | 72 (56%) | ＜0.001* |
| smooth | 67 (71%) | 57 (44%) |  |
| Blood flow degree |  |  |  |
| none | 79 (84%) | 102 (79%) | 0.111 |
| sparse | 15 (16%) | 21 (16%) |  |
| abundant | 0 (0%) | 6 (5%) |  |
| Blood flow pattern |  |  |  |
| none | 79 (84%) | 102 (79%) | 0.377 |
| dot | 9 (10 | 15 (12%) |  |
| single | 6 (6%) | 8 (6%) |  |
| branch-like | 0 (0%) | 4 (3%) |  |
| Gallbladder wall thickness (cm) | 0.30 (0.20-0.30) | 0.30 (0.30-0.30) | 0.309 |
| Gallbladder wall thickness types |  |  |  |
| normal | 85 (90%) | 119 (92%) | 0.855 |
| local thickening of the polyp attachment site | 2 (2%) | 3 (2%) |  |
| diffuse thickening | 7 (7%) | 7 (5%) |  |
| Gallstones | 7 (7%) | 10 (8%) | ＞0.999 |

Table S10. Error analysis in strategy LLMs-Text for Claude 3.5 Sonnet

|  | Correct cases (n=79) | Error cases (n=144) | *P* Value |
| --- | --- | --- | --- |
| Number of polyps |  |  |  |
| multiple | 44 (56%) | 84 (58%) | 0.777 |
| single | 35 (44%) | 60 (42%) |  |
| Size of the largest polyp (cm) | 1.10 (1.00-1.40) | 1.30 (1.13-1.58) | ＜0.001* |
| Echogenicity |  |  |  |
| hypoecho | 10 (13%) | 30 (21%) | 0.026* |
| isoecho | 31 (39%) | 70 (49%) |  |
| hyperecho | 38 (48%) | 44 (31%) |  |
| Echo uniformity |  |  |  |
| yes | 61 (77%) | 74 (51%) | ＜0.001* |
| no | 18 (23%) | 70 (49%) |  |
| Comet tail sign | 1 (1%) | 1 (1%) | ＞0.999 |
| Cauliflower shape | 9 (11%) | 34 (24%) | 0.033* |
| Base type |  |  |  |
| pedunculated | 53 (67%) | 101 (70%) | 0.652 |
| sessile | 26 (33%) | 43 (30%) |  |
| Polyp morphology ratio | 0.61 (0.39-1.00) | 0.52 (0.36-1.00) | 0.304 |
| Edge |  |  |  |
| rough | 24 (30%) | 75 (52%) | 0.002* |
| smooth | 55 (70%) | 69 (48%) |  |
| Blood flow degree |  |  |  |
| none | 69 (87%) | 112 (78%) | 0.086 |
| sparse | 10 (13%) | 26 (18%) |  |
| abundant | 0 (0.0%) | 6 (4%) |  |
| Blood flow pattern |  |  |  |
| none | 69 (87%) | 112 (78%) | 0.268 |
| dot | 7 (9%) | 17 (12%) |  |
| single | 2 (3%) | 12 (8%) |  |
| branch-like | 1 (1%) | 3 (2%) |  |
| Gallbladder wall thickness (cm) | 0.30 (0.20-0.30) | 0.30 (0.20-0.30) | 0.663 |
| Gallbladder wall thickness types |  |  |  |
| normal | 73 (92%) | 131 (91%) | 0.923 |
| local thickening of the polyp attachment site | 2 (2%) | 3 (2%) |  |
| diffuse thickening | 4 (5%) | 10 (7%) |  |
| Gallstones | 4 (5%) | 13 (9%) | 0.311 |


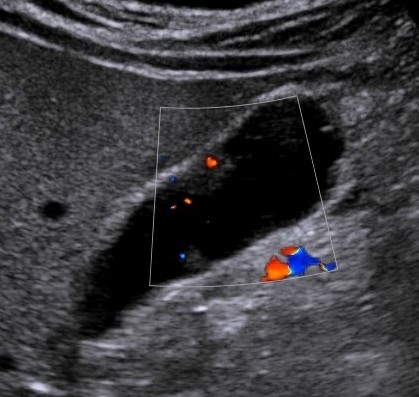


Example No.1. An example of dot and sparse blood flow (See Multimedia Appendix 2 for a higher resolution version.)


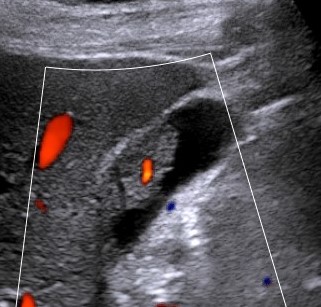


Example No.2. An example of single and sparse blood flow (See Multimedia Appendix 3 for a higher resolution version.)


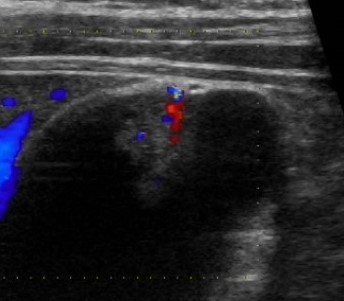


Example No.3. An example of branch-like and abundant blood flow (See Multimedia Appendix 4 for a higher resolution version.)


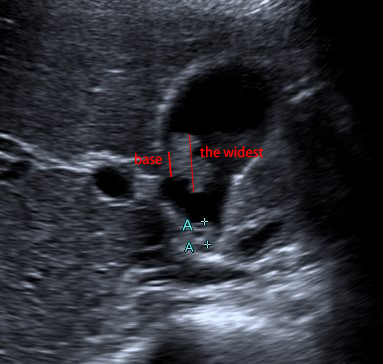


Example No.4. An example of a pedunculated polyp (See Multimedia Appendix 5 for a higher resolution version.)


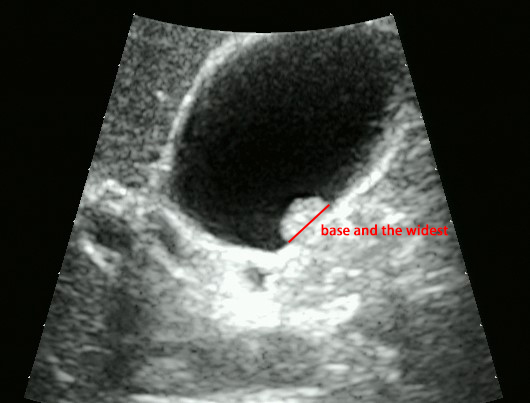


Example No.5. An example of a sessile polyp (See Multimedia Appendix 6 for a higher resolution version.)
