# Supplementary figures and images for: Evaluating Multiple Input Strategies of Large Language Models for Gallbladder Polyps on Ultrasound: Comparative Study
Source: JMIR Med Inform. 2025 Dec 23;13:e71178. doi: 10.2196/71178 (PMC12777648; doi:10.2196/71178)

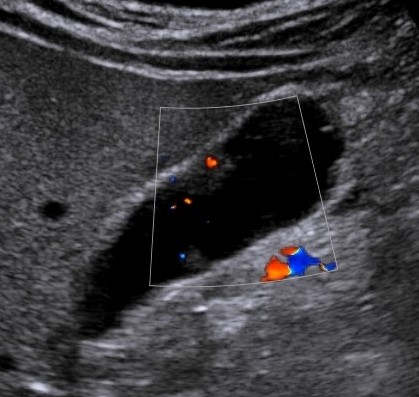

Supplement: Multimedia Appendix 2 [file medinform_v13i1e71178_app2.png]

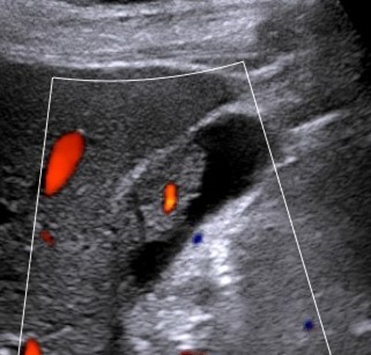

Supplement: Multimedia Appendix 3 [file medinform_v13i1e71178_app3.png]

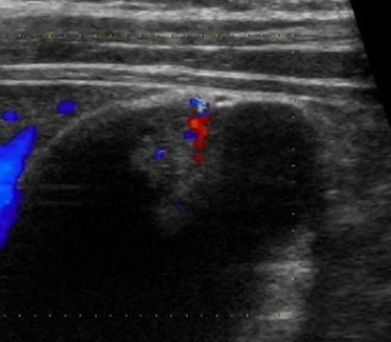

Supplement: Multimedia Appendix 4 [file medinform_v13i1e71178_app4.png]

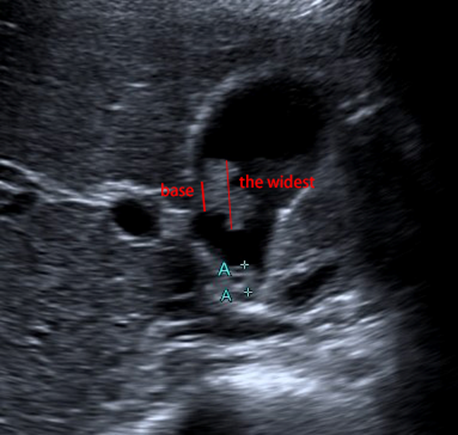

Supplement: Multimedia Appendix 5 [file medinform_v13i1e71178_app5.png]

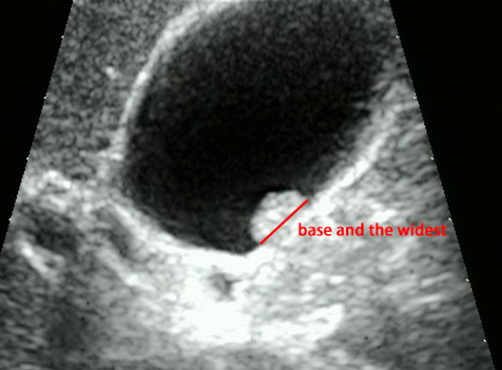

Supplement: Multimedia Appendix 6 [file medinform_v13i1e71178_app6.png]
